# Inflammation-Related Long Non-Coding RNA Signature Predicts the Prognosis of Gastric Carcinoma

**DOI:** 10.3389/fgene.2021.736766

**Published:** 2021-11-08

**Authors:** ShuQiao Zhang, XinYu Li, ChunZhi Tang, WeiHong Kuang

**Affiliations:** ^1^ First Affiliated Hospital of Guangzhou University of Chinese Medicine, Guangzhou University of Chinese Medicine, Guangzhou, China; ^2^ Medical College of Acupuncture-Moxibustion and Rehabilitation, Guangzhou University of Chinese Medicine, Guangzhou, China; ^3^ Guangdong Key Laboratory for Research and Development of Natural Drugs, School of Pharmacy, Guangdong Medical University, Dongguan, China

**Keywords:** gastric carcinoma (GC), inflammation, lncRNAs, long non-coding RNAs, prognosis (carcinoma), immune infiltration, signature

## Abstract

**Background:** Gastric carcinoma (GC) is a molecularly and phenotypically highly heterogeneous disease, making the prognostic prediction challenging. On the other hand, Inflammation as part of the active cross-talk between the tumor and the host in the tumor or its microenvironment could affect prognosis.

**Method:** We established a prognostic multi lncRNAs signature that could better predict the prognosis of GC patients based on inflammation-related differentially expressed lncRNAs in GC.

**Results:** We identified 10 differently expressed lncRNAs related to inflammation associated with GC prognosis. Kaplan-Meier survival analysis demonstrated that high-risk inflammation-related lncRNAs signature was related to poor prognosis of GC. Moreover, the inflammation-related lncRNAs signature had an AUC of 0.788, proving their utility in predicting GC prognosis. Indeed, our risk signature is more precise in predicting the prognosis of GC patients than traditional clinicopathological manifestations. Immune and tumor-related pathways for individuals in the low and high-risk groups were further revealed by GSEA. Moreover, TCGA based analysis revealed significant differences in HLA, MHC class-I, cytolytic activity, parainflammation, co-stimulation of APC, type II INF response, and type I INF response between the two risk groups. Immune checkpoints revealed CD86, TNFSF18, CD200, and LAIR1 were differently expressed between lowand high-risk groups.

**Conclusion:** A novel inflammation-related lncRNAs (AC015660.1, LINC01094, AL512506.1, AC124067.2, AC016737.1, AL136115.1, AP000695.1, AC104695.3, LINC00449, AC090772.1) signature may provide insight into the new therapies and prognosis prediction for GC patients.

## Introduction

Gastric carcinoma (GC) is a highly lethal and aggressive cancer, the third most common cause of cancer death globally, a disease with high molecular and phenotypic heterogeneity (Smyth et al., 2020), with over 1 million estimated new cases annually (Hacker and Lordick, 2015). *Helicobacter pylori* (HP) infection, age, high salt intake, and diets low in fruit and vegetables are significant risk factors for GC progression (Smyth et al., 2020). Although combined treatment, including surgery, chemo-radiotherapy, and chemotherapy, have shown remarkable improvements, the prognosis of GC remains undesirable (Shah and Kelsen, 2010; Cristescu et al., 2015). Moreover, the current TNM staging system of the American Joint Committee on Cancer (AJCC) or the Joint International Committee on Cancer (UICC) has shown valuable but insufficient prognostication for the prognosis and estimation of subsets of GC patients (Marano et al., 2015; Kim et al., 2016). Therefore, new biomarkers are needed to discriminate high-risk patients with GC to improve personalized cancer treatment.

The study of tumor-associated inflammation has increased rapidly in the past few decades, and it has also been described as a hallmark of cancer (Hanahan and Weinberg, 2011). Many cancers arise from irritation, chronic infection, and Inflammation, and the tumor microenvironment is mainly coordinated by inflammatory cells, which are indispensable players in fostering proliferation, neoplastic process, survival, and migration. Inflammation as an opportunity for anti-cancer therapies is on the rise. At the same time, long non-coding RNAs (lncRNAs) are a subset of non-coding RNA molecules with about 200 nt, which regulates gene expression and participates in various biological regulatory processes, including the regulatory process related to tumor genesis, progression, and metastasis (Gupta et al., 2010). At present, functional lncRNAs are considered critical roles in several biological regulatory processes, such as cell growth, development, angiogenesis, and inflammation (Ng et al., 2013; Li et al., 2014; Michalik et al., 2014; Ghazal et al., 2015). One recent study revealed that lincRNA-Cox2 and lncRNA NEAT1 promotes IL-6, an inflammatory cytokine, expression via distinct mechanisms. LncRNA NEAT1 acts further upstream and potentiates IL-6 expression by promoting the JNK1/2 and ERK1/2 signaling cascades (Ma et al., 2020). In a related study, Zhou et al. (2016) found that lncRNA ANRIL is involved in TNF-α-NF-κB signaling to regulate the inflammatory response in endothelial cells. Nevertheless, to date, serial studies that systematically evaluate inflammation-related lncRNA prognostic signature and its correlation with GC patients remain scarce.

In our study, a prognostic multi lncRNA signature of inflammation-related differentially expressed lncRNAs based on the Cancer Genome Atlas (TCGA) data was established for the first time. Then, we investigated the role of inflammation-related mRNA, immune responses, and N6-methylated- adenosine (m6A) mRNA status in GC prognosis.

## Methods

### Data Collection

RNA-sequence (32 normal and 375 tumor) data of 443 patients were extracted from the TCGA-STAD database. Clinical characteristics of the GC patients used in this study are shown in Table 1. The corresponding inflammation-related genes were downloaded from The Molecular Signatures database (MSigDB, http://www.gsea-msigdb.org/gsea/login.jsp) (Liberzon et al., 2015), a collection of annotated gene sets for use with Gene Set Enrichment Analysis (GSEA) software, which provided comprehensive and gene sets for inflammatory markers. Overall, we identified 561 inflammation-related genes (Supplementary Table S1). The association between lncRNAs in TCGA-LIHC dataset and inflammation-related genes from MSigDB was assessed by Pearson correlation analysis. If the correlation coefficient |*R* ^2^| was greater than 0.6 and *p* < 0.001, the correlation is considered significant, and then inflammation-related lncRNAs were selected. The clinicopathological data of GC patients were collected, including grade, age, stage, TMN, gender, survival time, and survival status. False discovery rate (FDR) < 0.05 and |log_2_FC| ≥ 1 were set as the significant differential expression of lncRNAs related to Inflammation. Firstly, we explored the functions of up and downregulated Inflammation related differentially expressed genes (DEGs). Then the biological processes, cellular components, and molecular functions associated with the DEGs were then evaluated by Gene Ontology (GO). Based on data from Kyoto Encyclopedia of Genes and Genomes (KEGG), the functions of biological pathways by different expressions of Inflammation-related lncRNAs were further analyzed in R software.

**TABLE 1:** The clinical characteristics of patients in the TCGA dataset.

Variable	Number of samples
Gender	
Male/Female	285/158
Age at diagnosis	
≤65/>65/NA	197/241/5
Grade	
G1/G2/G3/NA	12/159/263/9
Stage	
I/II/III/IV/NA	59/130/183/44/27
T	
T1/T2/T3/T4/NA	23/93/198/119/10
M	
M0/M1/NA	391/30/22
N	
N0/N1/N2/N3/NA	132/119/85/88/19

### Construction of the Inflammation-Related lncRNAs Prognostic Signature

To construct a robust and stable prognostic, predictive signature, we first used univariate Cox regression and lasso regression analysis, and finally used multivariate Cox regression analysis to construct an inflammation-related lncRNA signature, and stratified them based on the risk score (βlncRNA1 × ExpressionlncRNA1 + βlncRNA2 × ExpressionlncRNA2 + βlncRNA3×ExpressionlncRNA3 +…+ βlncRNAn × ExpressionlncRNAn). The relevant risk score of each GC patient was also evaluated. The lncRNAs were divided into low-risk (<median number) and high-risk (≥median number) groups based on median scores.

### The Predictive Nomogram

To establish a clinically feasible method to predict the survival rate of GC patients, we set a prediction model using nomograms and considered the clinicopathological manifestations of GC patients. The nomogram predicted 1 -, 2 -, and 3-years overall survival (OS) of GC patients, and the statistical significance was based on multivariate analysis of OS rate. The predictive factors included risk model and grade, age, stage, TMN, gender.

### Immunity Analysis and Related Gene Expression

Meanwhile, the relative immune cell infiltration level of individual samples was quantified by single-sample gene set enrichment analysis (ssGSEA) (Yi et al., 2020) comparing the CIBERSORT (Newman et al., 2015; Charoentong et al., 2017), CIBERSORT−ABS (Wang et al., 2020), QUANTISEQ (Plattner et al., 2020), MCPCOUNTER (Shi et al., 2020a), XCELL (Aran et al., 2017), EPIC (Racle et al., 2017) and TIMER (Li et al., 2017a)algorithms to evaluate the cellular component or cellular immune response lncRNAs signature between the high-risk and low-risk groups based on inflammation correlation. Then, the heatmap demonstrates the differences in immune responses under different algorithms. In addition, we used the ssGSEA method to quantitatively analyze the tumor-infiltrating immune cell subsets in the high-risk and low-risk group and evaluate their immune functions.

### Statistical Analysis

All the statistical analyses of data were performed in R software (version 4.0.3) with Bioconductor packages including “limma,” “survival,” “survminer.” Non paired *t*-test and Wilcoxon test were used to analyze the normal distribution and non-normal distribution variables. Based on FDR, the different expression of lncRNAs was corrected by the Benjamin Hochberg method to control the elevated false-positive rate. To analyze the GC DEGs associated immune status in each sample in the TCGA cohort, the relative infiltration of 20 immune cell types in the tumor microenvironment was calculated via ssGSEA with the application of the“GSVA” package in R. The prognostic performance of the GC predictive signature compared with other clinicopathological features was measured using the time-dependent receiver operating characteristic (ROC) and the decision curve analysis (DCA) (Vickers et al., 2008). Logistic regression analysis and a heatmap graph were used to analyze the association between inflammation-related lncRNAs and clinicopathological manifestations. Kaplan Meier survival analysis was used to evaluate the OS of GC patients based on Inflammation-related lncRNA signature. For each analysis, *p* < 0.05 in the results was considered statistically significant.

## Results

### Enrichment Analysis of Inflammation-Related Genes

**FIGURE 1:**
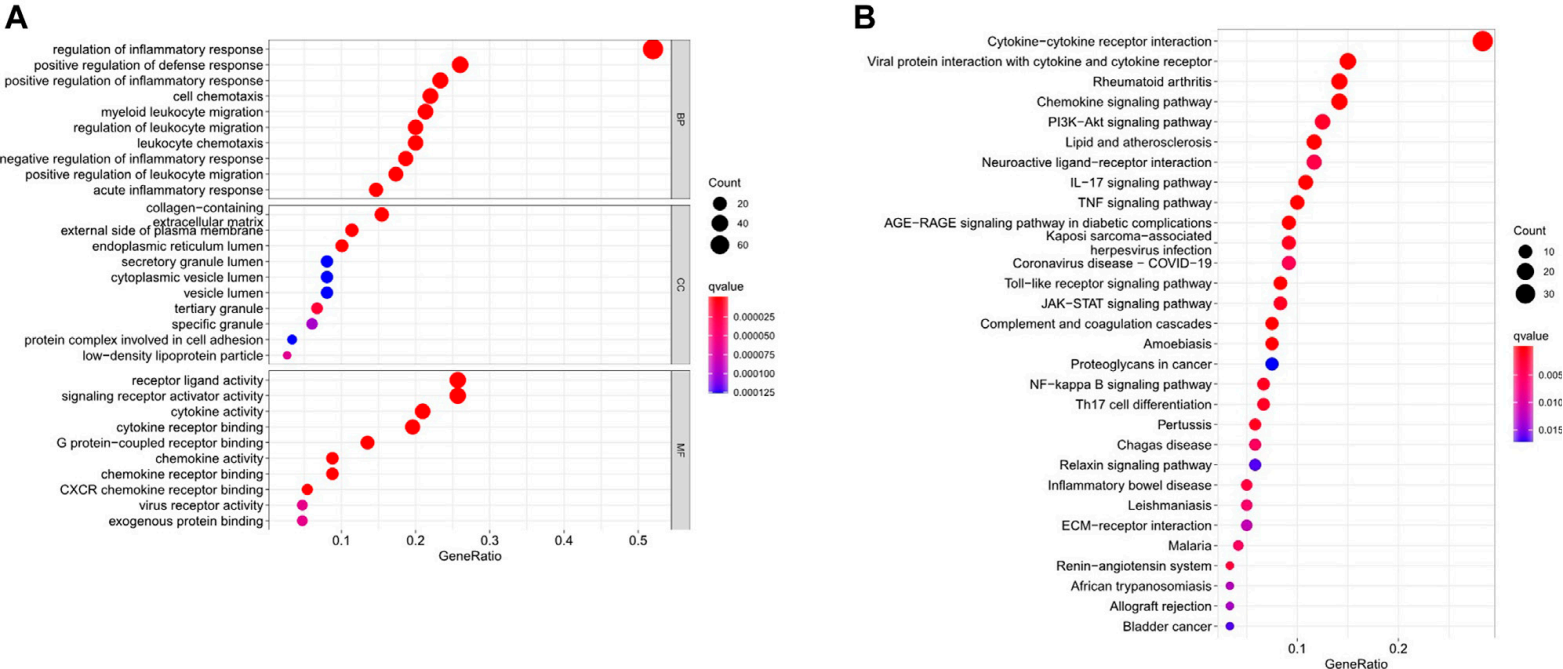
Go and KEGG analysis of differentially expressed inflammation-related genes. **(A)** Biological processes, molecular functions, and cellular components analysis of GO **(B)** Pathway enrichment analysis of KEGG.

We uncovered 150 inflammation-related DEGs (114 downregulated and 36 upregulated; Supplementary Table S2). Biological processes participated in the inflammatory response, defense response, and immune system process, among others. Molecular functions mainly regulated receptor ligand and signaling receptor activator activity, cytokine activity, and chemokine activity. Cellular components were regulated primarily on the collagen-containing extracellular matrix, external side of the plasma membrane, and endoplasmic reticulum lumen. Furthermore, KEGG analysis showed that the differentailly expressed genes were significantly enriched in Cytokine-cytokine receptor interaction, IL-17 signaling pathway, PI3K-AKT signaling pathway, TNF signaling pathway, JAK-STAT signaling pathway, Toll-like receptor signaling pathway, Chemokine signaling pathway, and NF-kappa B signaling pathway (Figure 1).

### The Inflammation-Based lncRNAs Prognostic Signature

**FIGURE 2:**
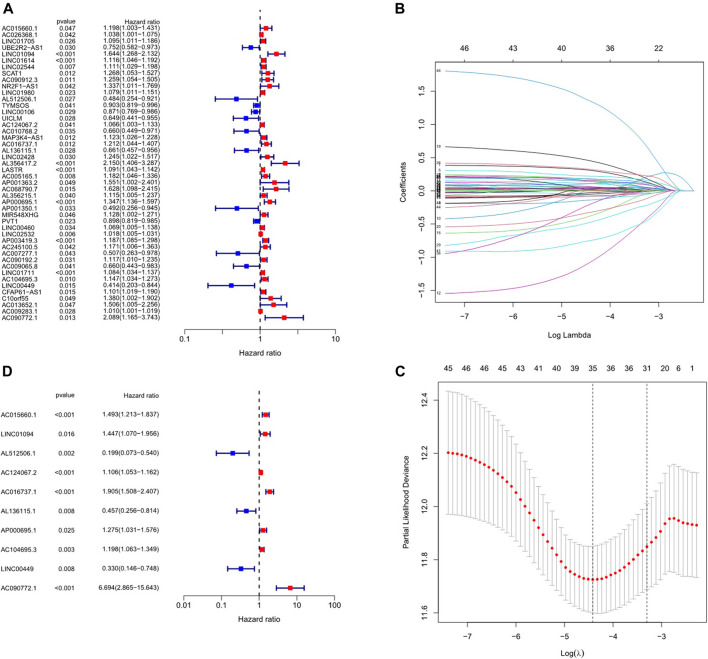
Construction of inflammation-related lncRNAs prognostic signature. **(A)** Univariate cox regression illustrated 46 inflammation-related lncRNAs associated with prognosis. **(B, C)** Inflammation-related lncRNAs with *p* < 0.05 in univariate Cox analysis were penalized by LASSO regression analysis. **(D)** Multivariate cox regression analysis revealed 10 inflammation-related lncRNAs prognostic signature of GC.

In the preliminary screening, 46 inflammation-related lncRNAs were obtained to be associated with OS by univariate Cox analysis (Figure 2A). Next, inflammation-related lncRNAs with *p* < 0.05 in univariate Cox analysis were penalized by Lasso regression (Figures 2B,C). Finally, 10 inflammation-related lncRNAs signature were found to be independent prognostic factors of GC patients by multivariate Cox regression analysis. (Figure 2D). A novel risk score was calculated by multiplying the lncRNA expression of each lncRNA and its corresponding coefficient, which was obtained by multivariate Cox regression analysis. The formula of the risk score was as follows: risk score = (Coefficient lncRNA1 × expression of lncRNA1) + (Coefficient lncRNA2 × expression of lncRNA2) + + (Coefficient lncRNAn × expression lncRNAn). Overall, we evaluated the risk score and identified 10 differently expressed inflammation-related lncRNAs as independent prognosis predictors of GC (Supplementary Table S3).

### Survival Results and Multivariate Examination

**FIGURE 3:**
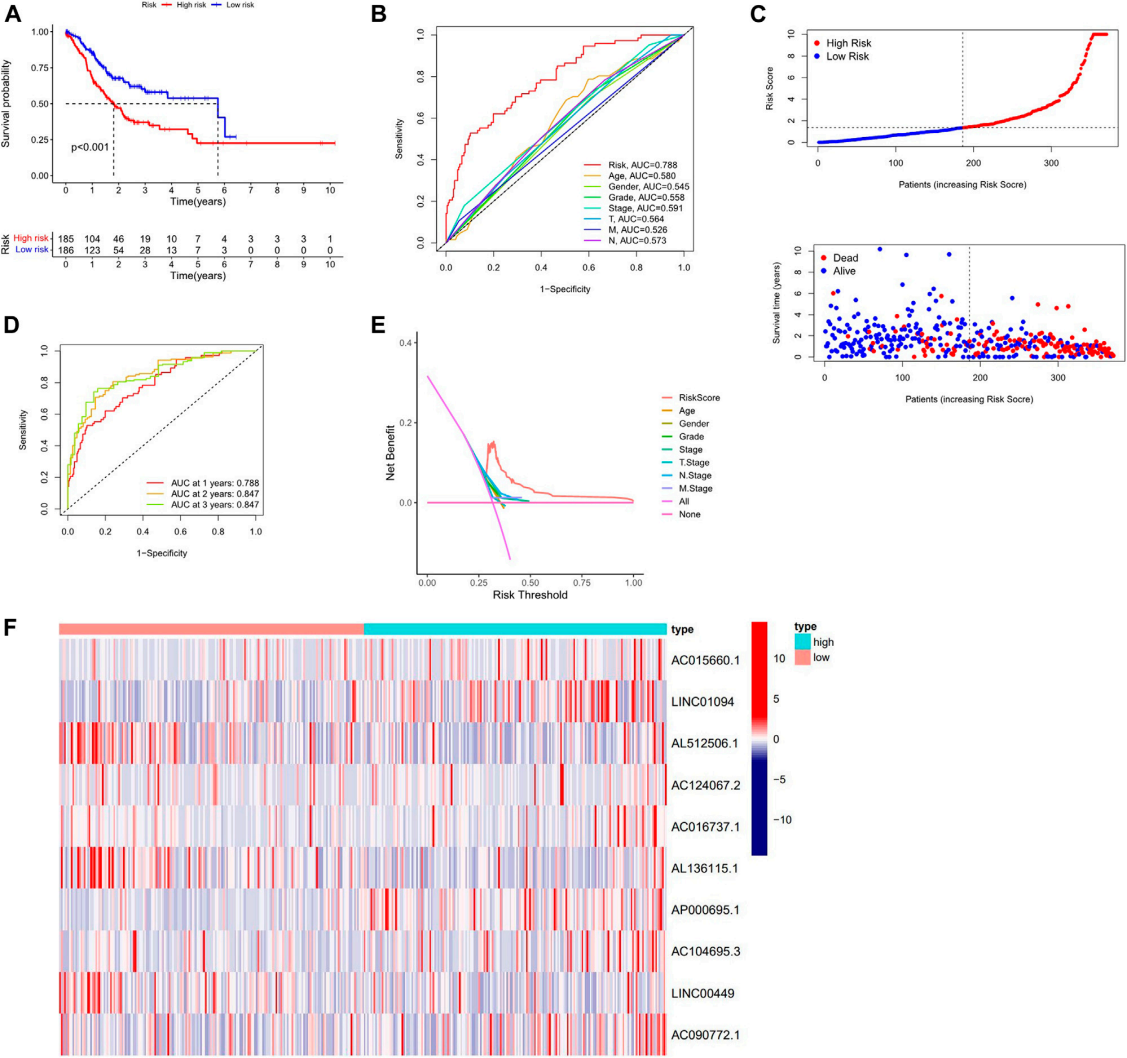
Validation of the Inflammation-related lncRNAs signature. **(A)** Survival curves result, **(B)**. The AUC values for forecasting OS based on risk factors, **(C)**. Risk score distribution and survival status of GC patients, **(D)** AUC of time-dependent ROC curves represented of 1, 2, 3-years survival rates of GC and risk score **(E)** The DCA plot. **(F)** Expression profile heatmap of the 10 inflammation-related lncRNAs signature.

**FIGURE 4:**
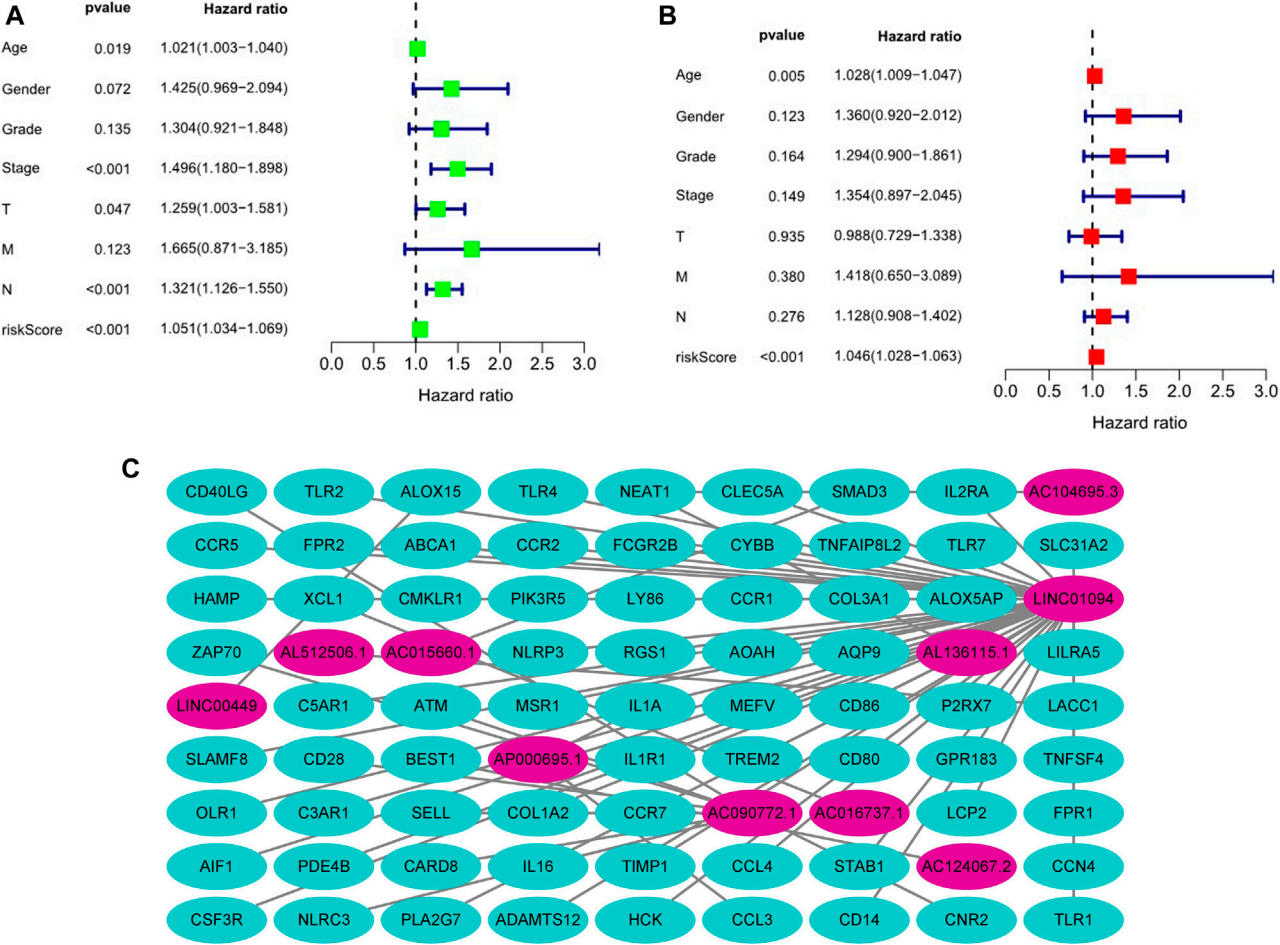
Estimation of the clinical value of the novel inflammation-related lncRNAs prognostic signature in GC patients based on univariate and multivariate COX analysis. **(A)**. Univariate Cox analysis, **(B)**. Multivariate Cox analysis, **(C)**. The lncRNA-mRNA relationship expression.

**FIGURE 5:**
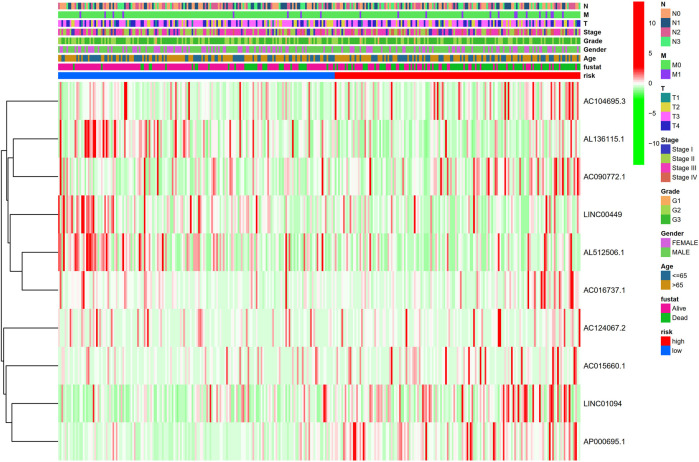
Heatmap for clinical pathology manifestations and inflammation-related lncRNAs prognostic signature.

**FIGURE 6:**
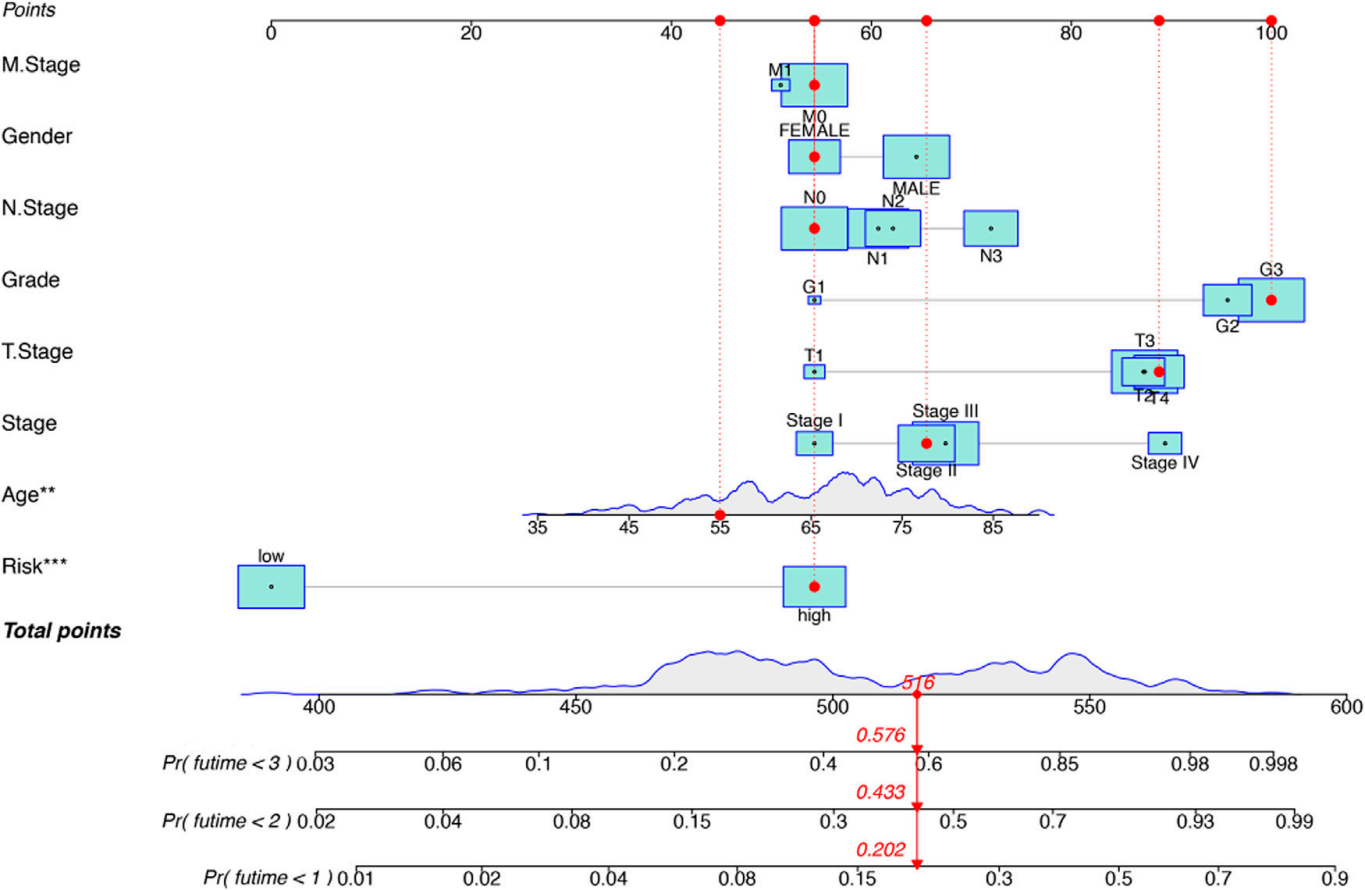
Construction of a hybrid nomogram based on both prognostic inflammation-related lncRNAs and prognostic-related clinicopathological factors.

Kaplan-Meier analysis revealed that GC patients in the high-risk group had poorer survival rates than those in the low-risk group (Figure 3A). Meanwhile, the signature lncRNAs, with an AUC of 0.788, exhibited superior performance in predicting GC prognosis than traditional clinicopathological features (Figures 3B,E). We used the patients’ risk survival status plots and found that the patients’ risk scores were inversely related to the GC patients’ survival. The heatmap revealed that the novel inflammation-related lncRNAs identified in our study were positively correlated with the risk model. (Figure 3C). The AUC for ROC analysis was 0.788, 0.847, and 0.847 for the predictive value of the 10 inflammation-related lncRNA signatures of GC patients for 1 -, 2 -, 3-years survival, respectively, (Figure 3D). Besides, the DCA plot indicated a robust and stable prognostic-predictive ability of the inflammation-related lncRNAs signature (Figure 3E). The heatmap demonstrated the difference in the expression of ten inflammation-related lncRNAs in the two risk population groups (Figure 3F). Univariate and multivariate Cox analysis revealed that the novel lncRNAs signature (HR: 1.05, 95CI: 1.03–1.07) was an independent prognosis predictor of GC patients (Figures 4A,B). The lncRNA-mRNA relationship was shown in the network (Figure 4C). Also, the heatmap analyzed the correlation between the novel prognostic signature and clinical-pathological features (Figure 5). The nomogram, which combined both the novel prognostic signature and the clinicopathological characteristics (Figure 6), was accurate and stable and thus applied to the clinical management for GC patients.

### Gene Set Enrichment Analyses

**FIGURE 7:**
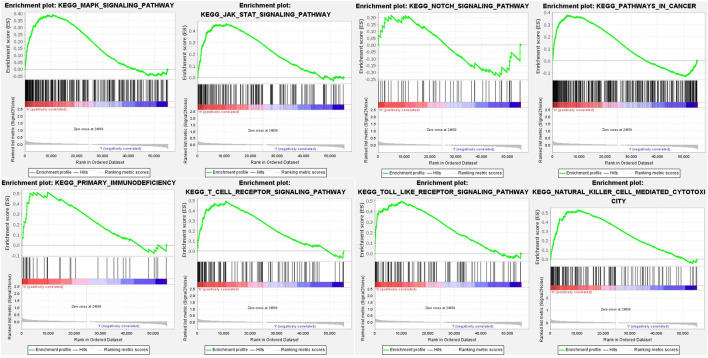
GSEA for inflammation-related lncRNAs signature.

To investigate the underlying pathways and biological processes, GSEA was used to reveal the significant pathways by which novel inflammation-related lncRNA signatures regulate tumor-associated and immune, such as natural killer cell-mediated cytotoxicity, primary immunodeficiency, T cell receptor signaling pathway, Toll-like receptor signaling pathway, MAPK signaling pathway, Notch signaling pathway, JAK-STAT signaling pathway and pathways in cancer (Figure 7; Supplementary Table S4).

### Immunological Reaction and Related Gene Expression

**FIGURE 8:**
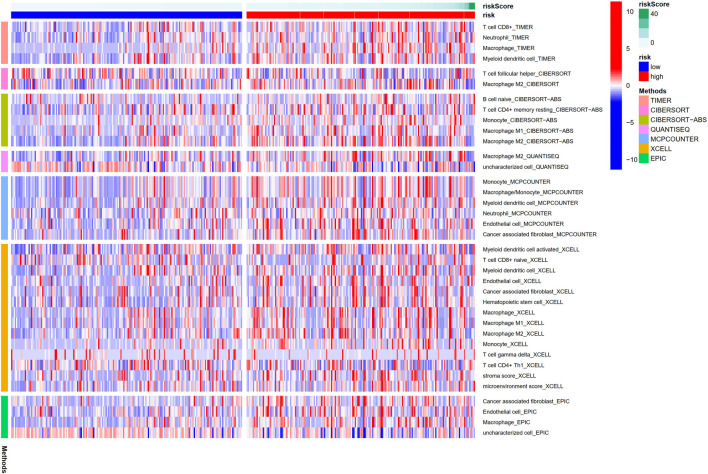
The immune responses among the high and low-risk group.

**FIGURE 9:**
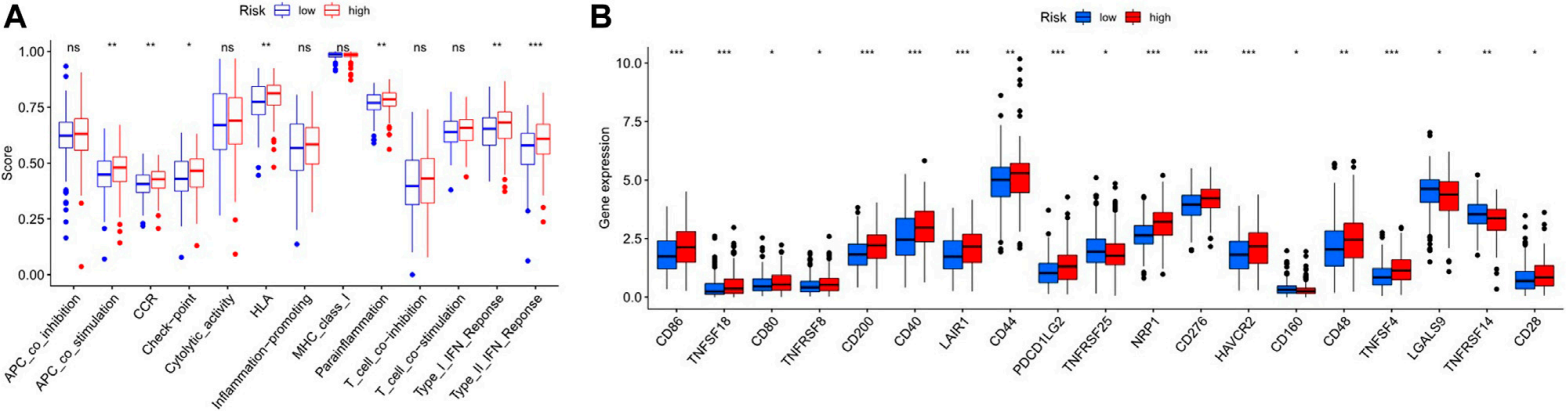
**(A)**. Correlation of immune cell subsets with related functions **(B)**. Comparison of immune checkpoints among the two GC risk groups.

**FIGURE 10:**
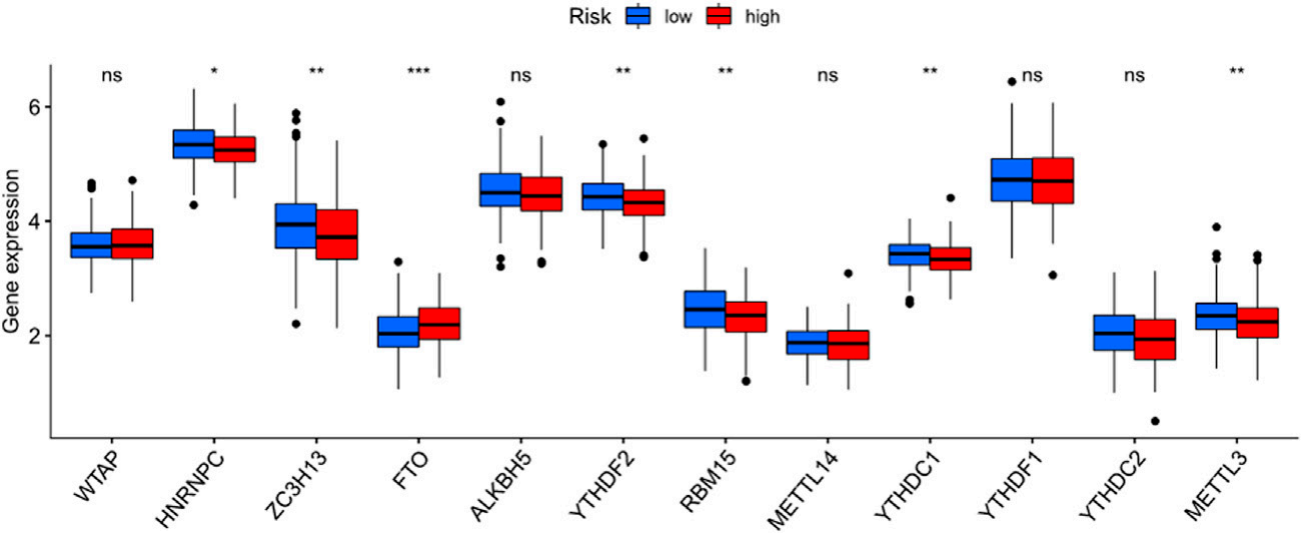
Comparison of m6A-related genes expression among the two GC risk group.

The heatmap showed the immunological responses based on QUANTISEQ, CIBERSORT, CIBERSORT-ABS, MCPCOUNTER, XCELL, TIMER, and EPIC algorithms. (Figure 8). ssGSEA correlation analysis of immune cell subsets with relevant functions based on TCGA-STAD data showed cytolytic activity, MHC class-I, HLA, type I INF response, type II INF response, parainflammation, and co-stimulation of APC differed significantly between the high and low-risk groups (Figure 9A). Given the significance of checkpoint inhibitor-based immunotherapy, differences in immune checkpoint expression among high-risk and low-risk groups were further explored. Among two risk groups, significant differences were discovered in gene expression, including CD86, TNFSF18, CD200, and LAIR1 (Figure 9B). Comparison of m6A-related mRNA expression among high- and low-risk groups revealed the significant expression of HNRNPC, RBM15, YTHDC1, YTHDF2, FTO, ZC3H13, and METTL3 (Figure 10).

## Discussion

Inflammation is of essentiality in the development of cancer because chronic Inflammation has been shown to increase cancer risk by causing tumor initiation, promotion, and metastatic progression (So et al., 2020). Meanwhile, one key hallmark of cancer is the ability of cancer cells to evade immune destruction (Hanahan and Weinberg, 2011). Thus, it has the potential to become a novel tumor therapy. In our study, a novel inflammation-related prognostic lncRNA signature was first constructed based on the existing clinical characteristic of GC patients from the TCGA dataset. Then, we investigated the potential roles of immune infiltrating cells and immune checkpoint inhibitors in the tumor microenvironment in GC prognosis. The findings of our study revealed potential biomarkers and therapeutic targets in the inflammation signaling pathways. Overall, our analyses uncovered 150 inflammation-related DEGs. KEGG analysis further indicated that these genes were mainly involved in the IL-17 signaling pathway, PI3K-Akt signaling pathway, Toll-like receptor signaling pathway, TNF signaling pathway, and JAK-STAT signaling pathway, and so forth. Some recent studies found that Toll-like receptors play a vital role in the innate immune system, particularly in the inflammatory response (Takeda and Akira, 2004), and Dectin-1 could suppress Toll-like receptor four signals to protect against chronic liver disease in hepatic inflammatory cells (Seifert et al., 2015). Balkwill (2006) reported that one of the vital chemical mediators implicated in inflammation-related cancer is TNFα-α; it is involved in the promotion and progression of experimental and human cancers, and its pathways lead to the activation of NF-κKey to the B, and AP-1 transcription factor complex is the intracellular connection.

Blocking the link of inflammation to cancer might be potentially a new way for tumor treatment. Overall, in our study, the ten differently expressed inflammation-related lncRNA signature found was an independent prognostic predictor of GC. A recent study found LINC01094 could promote the proliferation, migration, invasion and epithelial-mesenchymal transition of ovarian cancer cells by adsorbing miR-577 (Xu et al., 2020). Besides, LINC01094 could promote clear cell renal cell carcinoma development through the miR-224-5p/CHSY1 regulatory axis (Jiang et al., 2020). Linc01094 promotes proliferation, migration and invasion of glioma cells by adsorbing miR-330-3p and upregulating MSI1 expression (Zhu et al., 2020). In a related study, overexpression of LINC00449 could inhibit acute myeloid leukemia cell invasion and cell proliferation *in vitro* and *in vivo*, and the LINC00449/miR-150/FOXD3 signaling pathway may be a novel prognostic biomarker or therapeutic target for the treatment of acute myeloid leukemia (Shi et al., 2020b). Thus, it is of importance to establish a novel prognostic multiinflammation-related lncRNAs signature for GC patients. Such findings may provide valuable insights for cancer control in the future.

The Inflammation-related lncRNAs with different expressions were divided into low-risk and high-risk groups to investigate their potential roles in GC. The Inflammation-related lncRNA signature of the high-risk group was correlated with poor prognosis of GC patients in the study results. Moreover, the inflammation-related lncRNA signature had an AUC of 0.788 but performed well in other validations such as DCA, demonstrating their utility in predicting GC prognosis and indicating that our risk signature also outperforms traditional clinic pathology characteristics.

The direct correlations between lncRNAs and cancer-derived Inflammation, epithelial-to-mesenchymal transition, metastasis, and other hallmarks of cancer indicate their potential as cancer biomarkers and targets (Tamang et al., 2019). LncRNA H19 could be induced by *helicobacter pylori* infection to promote gastric cancer cell growth via strengthening NF-κB-induced inflammation (Zhang et al., 2019). Besides, CCAT1 lncRNA Promotes Inflammatory bowel disease malignancy by destroying Intestinal Barrier via downregulating miR-185-3p (Ma et al., 2019). Increasing evidence suggests that lncRNA is critical in mediating both inflammation and cancer (Barsyte-Lovejoy et al., 2006; Guo et al., 2019; Song et al., 2020; Sun et al., 2020).

Our study, GSEA revealed immune and tumor-related pathways for individuals in the low- and high-risk groups. TCGA revealed significant differences in HLA, MHC class-I, cytolytic activity, parainflammation, co-stimulation of APC, type II INF response, and type I INF response between the low and high-risk groups. Moreover, Immune checkpoints such as CD86, TNFSF18, CD200, and LAIR1 were differently expressed between high and low-risk groups. Li et al. speculated that Inflammation triggered by lncRNA CRNDE could regulate tumorigenesis and development through the TLR pathway (Li et al., 2017b). In recent years, lncRNAs have gained attention as essential regulators of gene expression acting through versatile interactions with DNA, RNA, or proteins. Intriguingly, lncRNAs play crucial roles in modulating innate immune cell development and inflammatory gene expression (Elling et al., 2016). Activating inflammatory pathways, particularly the IFN response, can sensitize animal cancer models and cancer patients to immune checkpoint inhibitors and positively contribute to anti-tumor activity (Zemek et al., 2019). At present, few studies have explored the relationship among inflammation and immune checkpoint inhibitors. Therefore, lncRNAs might be critical players in the transformation of inflammation to cancer through the immune microenvironment.

Although we identified a predictive risk model with ten inflammation-related lncRNAs and confirmed that the risk model was significantly associated with Inflammation, this work has limitations. The study conducted with bioinformatics analysis was not robust enough and needed to be confirmed via experimental verification. Hence, further laboratory experiments, including a multicenter study with larger sample sizes, are required. In summary, in this study, we developed a robust prognostic risk model with ten inflammation-related lncRNAs. Compared to other clinical parameters, the risk score is an independent prognostic index. Therefore, this risk model may serve as a prognostic signature and provide clues for individualized immunotherapy for GC patients.

## Conclusion

Specific Inflammation-related lncRNAs can provide individualized prediction of GC patients’ prognosis.

## Data Availability

The original contributions presented in the study are included in the article/Supplementary Material, further inquiries can be directed to the corresponding authors.

## Abbreviation

GC, Gastric carcinoma; OS, overall survival (OS); DEGs, differentially expressed genes; TCGA, The Cancer Genome Atlas; GO, Gene ontology; KEGG, Kyoto Encyclopedia of Genes and Genomes; GSEA, Gene set enrichment analyses; FDR, false discovery rate; ssGSEA, single-sample gene set enrichment analysis (ssGSEA).
